# Unresolved issues in the diagnosis and management of thrombotic antiphospholipid syndrome

**DOI:** 10.1016/j.rpth.2025.102724

**Published:** 2025-03-07

**Authors:** Deepa J. Arachchillage, Mike Laffan

**Affiliations:** 1 Centre for Haematology, Department of Immunology and Inflammation, Imperial College London, London, United Kingdom; 2 Department of Haematology, Imperial College Healthcare NHS Trust, London, United Kingdom

**Keywords:** antiphospholipid syndrome, immunomodulation, stroke, thrombosis, vitamin K antagonist

## Abstract

Antiphospholipid syndrome (APS) is a highly prothrombotic autoimmune disease characterized by the persistent presence of antiphospholipid autoantibodies (aPL) in association with thrombotic or nonthrombotic macro- and microvascular manifestations and/or pregnancy complications. This review is restricted to thrombotic APS. Since the publication of the American College of Rheumatology/European Alliance of Associations for Rheumatology classification criteria for APS, several authors have emphasized the difference between “classification” and “diagnosis” as a potential pitfall for clinicians. In addition to challenges associated with the diagnosis of APS, there are many unresolved areas in understanding pathogenesis and in the management of both thrombotic and obstetric APS. Although APS is an antibody-mediated autoimmune disease, secondary thrombosis prevention is achieved by anticoagulation, mainly with vitamin K antagonists, such as warfarin, rather than immunomodulation. Evidence is convincing for the use of vitamin K antagonists in triple-positive APS with venous thromboembolism. However, the best anticoagulant approach in the management of venous thromboembolism patients with single or dual positive aPL is not clear. Management of patients with stroke or arterial thrombosis with aPL remains a major unresolved issue, although some guidelines recommend the use of warfarin rather than antiplatelet therapy as the first-line treatment of stroke in APS. Recurrent thrombosis, despite therapeutic anticoagulation, remains a frequent problem and may be explained by the contribution of thrombo-inflammation in patients with thrombotic APS. In this narrative review, we discuss some of the unresolved issues in the diagnosis and management of thrombotic APS.

## 1 Introduction

Antiphospholipid syndrome (APS) is a highly prothrombotic autoimmune disease characterized by the persistent presence of antiphospholipid autoantibodies (aPL) in association with thrombotic macro- and microvascular manifestations and/or pregnancy complications. The aPL included in the current diagnosis of APS include lupus anticoagulant (LA), immunoglobulin (Ig) G or IgM anticardiolipin (aCL), and anti-β2-glycoprotein I (β2GPI) antibodies; however, there is a multitude of autoantibodies against diverse target proteins reported to be present in patients with APS which may contribute to its pathogenesis. Although macrovascular thrombosis is the hallmark of the disease, and veins are more frequently affected than arteries, thrombosis can occur in any organ or tissue in the body, affecting both macro and microvascular circulations. Deep vein thrombosis/pulmonary embolism is the most common manifestation overall, while stroke and transient ischemic attack are the most frequent arterial thromboses. A small proportion of patients with APS present with thrombosis at unusual sites such as cerebral venous sinus thrombosis, retinal vein or artery thrombosis, and intra-abdominal thrombosis. Microvascular thrombosis may occur in any organ system in APS and can be found in up to 12% of patients [1]. The kidneys (nephropathy) and skin (chronic ulcers and livedoid vasculopathy) seem to be the most frequently affected by microvascular thrombosis [2].

Although the presence of aPL is essential for the development of pathogenic complications, thrombosis is intermittent, and not all individuals with aPL develop thrombosis, suggesting a second hit, such as infection or inflammation, is required [3]. This may also imply that not all aPL are equally pathogenic, even when they appear to have the same specificity. Furthermore, there is wide variation in the clinical presentation, and some individuals develop both macro- and microvascular thrombosis, although in the majority, it is macrovascular. We do not know if this is due to differences in antibodies, individual vascular variation, or the nature of “second hits.” Vitamin K antagonists (VKAs) are the only oral anticoagulants approved for use in thrombotic APS, especially those with triple-positive APS (presence of all 3 aPL included in the current diagnostic tests). However, previous studies demonstrated that despite VKA anticoagulation, the risk of recurrent thrombosis is 30% in 10 years [4] for aPL patients who are triple positive, demonstrating that our current treatment strategy with anticoagulation is not optimal for at least some patients.

Obstetric APS is considered the single most recognizable risk factor for recurrent pregnancy loss and late placenta-mediated obstetric complications [5]. Placental thrombosis, inflammation, and complement activation all play major roles in the pathogenesis of obstetric APS [6].

In this narrative review, we focus only on the unresolved issues related to the diagnosis and management of thrombotic APS, including catastrophic APS (CAPS), which is a rare (<1% of APS cases) but potentially life-threatening variant of APS characterized by multiple microvascular thromboses leading to multiorgan failure. We discuss pragmatic approaches in challenging scenarios where we do not have solid evidence to make firm conclusions.

## 2 Unsolved Issues in the Diagnosis of APS

### 2.1 Classification vs diagnosis

Although the history of APS can be traced to observations made during screening programs for syphilis conducted in the mid-20th century [7,8], there are no standardized diagnostic criteria in clinical practice. The first APS classification criteria were developed by a group of experts in 1999 [9], which included only LA and aCL IgG and IgM as laboratory criteria. Subsequently, the 2006 revised Sapporo classification added IgG and IgM anti-β2GPI [10]. Although classification criteria were developed to ensure uniform populations of patients for clinical/research studies, in practice, these classification criteria have been frequently adopted for the routine clinical diagnosis of APS. Consequently, since the recent publication of the American College of Rheumatology (ACR)/European Alliance of Associations for Rheumatology (EULAR) classification criteria for APS [11], several authors have emphasized the difference between “classification” and “diagnosis” as a potential pitfall for clinicians [12]. To some extent, this problem is inevitable because if the criteria are used as intended for clinical trials and diagnosis is a prelude to therapy, clinicians will tend to make the diagnosis in those who fulfill the classification criteria. The explicit aim of the EULAR/ACR classification was to maximize specificity, which for thrombotic APS will likely reduce the number of patients for whom the clinician has therapeutic data. The danger is that some patients may not be diagnosed and so not benefit from therapy, even though this will require some extrapolation. On the other hand, the scoring system will allow stratification, which may help refine therapy at an individual patient level. From a thrombosis point of view, they may also help define anticoagulation strategies for myocardial infarction and APS nephropathy for example. Finally, the classification will include other clinical phenomena associated with aPL, such as hematologic abnormalities (thrombocytopenia and hemolytic anemia) and cardiac valve abnormalities, which may improve understanding of phenotypes.

### 2.2 Identification of clinical features for thrombotic APS

In most cases, clinical identification is not contentious, and diagnosis of thrombosis is unambiguous. More difficult is determining whether there is sufficient existing thrombosis risk to make testing for aPL unnecessary; guidelines suggest not testing when there is a pre-existing high risk of thrombosis. The consequences of this strategy have not been tested in practice. Testing would be less cautious if the pathogenic implications of the results were clearer; that is to say, we understood which aPL confer the greatest thrombotic risk and why [12].

### 2.3 Standardization

EULAR/ACR requires that the aPL be detected by enzyme-linked immunosorbent assay, a condition that has been criticized [12]. Firstly, it implies that enzyme-linked immunosorbent assays give consistent results between kits and centers, which has been shown not to be the case. Secondly, the current practice in many laboratories has been to adopt automated methods for simplicity and internal consistency. This makes the use of numerical values for diagnosis and classification unsafe and is a barrier to risk stratification. The International Society on Thrombosis and Haemostasis (ISTH) Scientific and Standardization Committee (SSC) suggests that the use of locally determined normal ranges and a centile system for cutoff would be better in the absence of reference standards [13,14]. A recent report from the ISTH SSC evaluated the use of interval-specific likelihood ratios as a useful approach. This avoids the problem of strictly numerical comparisons, allows local normal ranges, and has more information than a simple positive-negative dichotomization [15]. In thrombotic APS, at least, this improved the posttest probability of APS and, interestingly, also raised uncertainty of the value of IgM antibodies in obstetric APS. Some form of standardization is a major outstanding problem affecting all aspects of APS and will be a barrier to the implementation of EULAR/ACR-based results.

### 2.4 Noncriteria antibodies

Multiple antibody specificities have been described in APS but are not included in the classification criteria. Nonetheless, for some, there is evidence of a pathogenic role. The most widely studied noncriteria antibodies are anti-phosphatidylserine/prothrombin antibodies (anti-PS/PT) that target the anionic phospholipid phosphatidylserine and the procoagulant plasma protein prothrombin and antibodies specifically against the N-terminus of the β2GPI molecule (domain 1 specific anti-β2GPI antibodies).

Anti-PS/PT IgM/IgG are found at higher frequency in patients with APS and show a strong association with diagnosis, particularly in the presence of LA. Indeed, it has been suggested that the anti-PS/PT antibody could serve as a substitute for LA detection, perhaps in patients on anticoagulants [16]. They may also define a second type of triple-positive patient, but their utility will be defined by their ability to predict thrombosis more accurately in APS patients or controls and/or to identify patients at risk of thrombosis who do not otherwise fulfill the classification criteria [17,18]. Although their predictive power is not established, they are reported to be widely incorporated in diagnosis, so it will be regrettable if they are not included in future clinical studies using the ACR/EULAR criteria [19].

A second unresolved question is why anti-D1 antibodies, for which there is a good pathophysiological model, remain a weaker predictor of thrombosis than the LA, which has no clear pathophysiological model [20,21]. Again, a more detailed understanding of antibody specificity and functional effects is required to inform diagnosis as well as management and to bridge the gap between diagnosis and classification. Overall, the problem of how to match the wide variety of aPL in APS to the wide variety of APS clinical phenotypes and apply this to personalized therapy remains unsolved.

## 3 Management of Persistently Positive aPL with or without Thrombosis

There are many unresolved areas in the management of individuals with persistently positive aPL with or without thrombosis. In this review, we have focused on several of them, which are summarized in the Table.

**Table:** Unresolved issues related to the management of thrombotic antiphospholipid syndrome.

• Role of DOACs in single or dual positive VTE
• Development of diagnostic criteria in routine clinical practice
• Management of arterial thrombosis – anticoagulation (VKA target INR: 2.0-3.0 or 3.0-4.0, or the role of DOACs) or antiplatelet treatment (single or dual antiplatelet treatment) or the combination of anticoagulation with antiplatelet treatment.
• How to manage recurrent venous or arterial thrombosis
• Role of immunomodulation
• Can we stop anticoagulation in patients who previously had positive aPL and thrombosis but now are negative?
• Management of asymptomatic carriers
• Diagnosis and management of catastrophic APS

aPL, antiphospholipid antibody; APS, antiphospholipid syndrome; DOAC, direct oral anticoagulant; INR, international normalized ratio; VKA, vitamin K antagonist; VTE, venous thromboembolism.

## **4** Management of Arterial Thrombosis in APS

An important unresolved question in the management of APS is whether arterial thrombosis in APS patients is best managed with antiplatelet agents (single or dual) alone, anticoagulant therapy alone, or by a combination of the anticoagulant and antiplatelet treatment. In general, the mainstay of treatment for thrombotic APS is initial anticoagulation with heparin and VKAs, such as warfarin, with a target international normalized ratio (INR) of 2.5 (2.0-3.0) [22]. This was based on the results of the 2 randomized controlled trials (RCTs) that showed high-intensity warfarin (INR, 3.0-4.0) was not superior to standard-intensity warfarin (INR, 2.0-3.0) for the secondary prevention of venous or arterial thrombosis [23,24]. However, these RCTs mainly included APS patients with venous thrombosis (76% [87/114] and 60% [65/109], respectively) [23,24]. The Antiphospholipid Antibodies and Stroke Study was a prospective cohort study comparing warfarin anticoagulation (INR range, 1.4-2.8) with aspirin (325 mg/d) in stroke prevention in patients considered to be APS [25]. However, patients included in this study were in their 60s with increased vascular risk factors, had tested for aPL only at study entry, and included IgA antibodies as laboratory diagnostic criteria. These limitations raise the possibility that some recruits may not have had APS. This study found no benefit of anticoagulation with warfarin over aspirin [25]. Well-designed RCTs for assessing the anticoagulant intensity with warfarin or anticoagulant vs antiplatelet treatment for APS patients with arterial thrombosis using standard classification criteria are lacking to date, and this question remains open.

A network meta-analysis of 13 studies (*n* = 719 patients) reported that the use of antiplatelet treatment and warfarin resulted in a significant reduction in the risk of recurrent arterial thrombosis compared with single antiplatelet therapy alone (relative risk, 0.41; 95% CI, 0.2-0.85), with no significant difference in bleeding rates [26]. This meta-analysis did not compare anticoagulants alone vs anticoagulant and antiplatelet treatment.

An ongoing ISTH SSC registry, “Augmented antithrombotic treatment regimens for patients with arterial thrombotic APS,” aims to assess the effects of VKA with an INR range of 2.0 to 3.0 with or without low-dose aspirin (LDA), VKA with an INR of 3.0 to 4.0, and dual antiplatelet treatment in APS patients with arterial thrombosis [27].

In the absence of clear evidence of efficacy and safety for a single antithrombotic regimen, current clinical practice for the management of stroke/arterial thrombosis in APS varies from VKAs with a target INR of 2.0 to 3.0 or 3.0 to 4.0, single or dual antiplatelet agents, or a combination of VKA and antiplatelet. Our practice is to anticoagulate with warfarin, aiming for a target INR of 2.5 (2.0-3.0) [28] in patients with stroke/arterial thrombosis [28,29] with a particular focus on vascular risk reductions, as the majority of patients have at least 1 vascular risk factor such as smoking, diabetes, hypertension, or hypercholesterolemia. We consider hydroxychloroquine (HCQ) as an adjunct (discussed in section 6 in detail in the role of immunomodulation in thrombotic APS) in triple-positive APS patients if there are no contraindications [29]. Additionally, the combination of aspirin and standard-intensity warfarin did not show a significant difference in bleeding compared with antiplatelet alone in the previously mentioned network meta-analysis of APS patients with arterial thrombosis [26]. However, there is no direct comparison of bleeding rates between VKAs with a target INR of 3.5 (3.0-4.0) and VKAs with a target INR of 2.5 (2.0-3.0) plus aspirin in patients with thrombotic APS. Direct oral anticoagulants (DOACs) are generally not recommended for arterial thrombosis– see section 5 [30,31].

## 5 Role of DOACs in Single or Dual Positive VTE

Following an RCT comparing warfarin vs rivaroxaban in patients with triple-positive thrombotic APS, which was prematurely terminated due to a high recurrence rate in the rivaroxaban arm [31], it was recommended that DOACs should not be used in patients with thrombotic APS, especially those who were triple positive [22,30,32]. A subsequent meta-analysis of all 4 open-labeled RCTs, including 472 patients comparing a VKA vs DOAC, showed that DOACs were associated with an increased risk of arterial thrombotic events (odds ratio [OR], 5.43; 95% CI, 1.87-15.75; *P* < .001), particularly stroke (OR, 10.74; 95% CI, 2.29-50.38) [33]. Furthermore, this risk remained high whether the index thrombotic event was venous or arterial [33]. However, there was no difference in the risk of recurrent venous thrombosis or major bleeding between patients treated with DOACs and warfarin [33]. In the predefined subgroup analysis, the association between DOAC and arterial thrombosis was significantly higher in patients with triple-positive aPL. However, there was only a trend toward a higher risk of recurrent arterial thrombosis for patients with single or dual positive aPL (OR, 4.29; 95% CI, 0.75-24.43) [33]. Interestingly, these DOAC/warfarin APS trials showed overall lower rates of recurrent thrombosis compared with previous studies with VKA alone, which may reflect a selection bias. In clinical practice, some patients remain on DOAC therapy for several years without recurrence of thrombosis, especially those with single or dual positive aPL profiles. Therefore, it remains an unresolved question as to the most appropriate anticoagulant strategy for patients with single or dual positive aPL. Our practice is to anticoagulate all confirmed APS patients with VTE with VKA (warfarin) irrespective of aPL profile, reserving DOACs only for those who cannot tolerate warfarin therapy or who do not wish to take warfarin despite understanding the current evidence [22,29]. However, it is always our practice to have an informed and shared decision with the patient regarding their management plan.

## 6 Management of Recurrent Thrombosis, Including Role of Immunomodulation

Recurrent thrombosis, despite adequate anticoagulation, remains a significant challenge in thrombotic APS [4]. A recent UK-wide study found that the risk of recurrent thrombosis is around 40% in all patients with APS [34]. There are no clinical trial data to guide management of patients with recurrent thrombosis despite being on therapeutic anticoagulation. Therefore, clinical approaches vary depending on expert opinion, case-based studies, and indirect evidence. It is a straightforward decision to switch to warfarin if the patient was on a DOAC when a recurrence occurred. Again, the target INR when switching to warfarin is debatable. Since all RCTs compared warfarin with a target INR of 2.5 (2.0-3.0) with a standard dose of DOAC, even in patients without APS, it is our clinical practice to aim for a target INR of 3.0 (2.5-3.5) if a thrombotic APS patient develops a recurrent VTE while on a DOAC.

For patients who developed recurrent thrombosis while on warfarin, the first step would be to establish that the INR was within the therapeutic range at the time of recurrence. Patients with positive LA should have their INR monitored using venous INR with a thromboplastin reagent with known insensitivity to LA. Point-of-care INR should be used only after a comparative exercise to assess whether venous INR and point-of-care INR differences are within 0.5 by repeating INRs simultaneously on at least 3 occasions [22,35]. If the only thromboplastin available is sensitive to LA, measuring amidolytic factor X activity is useful and should be around 20-30 IU/dL in a patient on warfarin with an INR of 2.5 (2.0-3.0) [36,37]. Once it is confirmed that the recurrent thrombosis occurred while on therapeutic INR, either increasing the target INR to 3.5 (3.0-4.0) with a short bridging period of treatment dose low-molecular-weight heparin (LMWH) until the target INR is achieved or adding an antiplatelet or immunomodulatory therapy while keeping the target INR at 2.5 (2.0-3.0) is suggested.

In the event of recurrent arterial thrombosis despite anticoagulation, we add an antiplatelet while keeping the same target INR or increase the target INR from 2.5 (2.0-3.0) to 3.5 (3.0-4.0) without adding antiplatelet treatment [28,29]. However, as platelets play a major role in the pathogenesis of arterial thrombosis, the former approach is frequently used. Alternative approaches considered by some clinicians would be parenteral anticoagulant treatments such as LMWH or fondaparinux [38]. However, daily subcutaneous injections are not generally a practical option, although in a small group of patients, it may be preferred. Furthermore, the standard dose of LMWH or fondaparinux may not be adequate in patients with recurrent thrombosis while on standard-intensity VKA, although additional beneficial effects such as anticomplement and anti-inflammatory effects of LMWH may be beneficial [39]. Finally, the long-term consequences of LMWH, such as osteopenia/osteoporosis, need to be considered [40,41], including monitoring with bone density scan and optimization of vitamin D/calcium.

APS is an antibody-mediated autoimmune disease, and increasing evidence suggests that thrombo-inflammation plays a major role in the pathogenesis of thrombosis [42]. Recurrent thrombosis, despite being on treatment dose anticoagulation, may reflect the role of thrombo-inflammation in patients with APS. Therefore, targeting the immune system using an immunomodulatory agent such as HCQ has been considered in patients with recurrent thrombosis despite adequate anticoagulation.

HCQ is a first-line treatment option for inflammatory rheumatic disorders, such as rheumatoid arthritis and systemic lupus erythematosus. Experimental models and clinical studies have demonstrated that HCQ has antithrombotic effects. We have demonstrated that HCQ can modulate the endothelial prothrombotic phenotype *in vitro,* such as the downregulation of tissue factor expression and reduced thrombin generation in the presence of inflammation [43]. Several small studies in patients with APS demonstrated the efficacy of HCQ in preventing recurrent thrombosis or provided evidence of a reduced prothrombotic phenotype in plasma. In a study of 22 patients with aPL, Schreiber et al. [44] reported that HCQ significantly reduced soluble tissue factor levels at 3 months compared with baseline. In a study of 40 patients nonrandomly assigned to receive a VKA alone (*n* = 20) and VKA plus HCQ (400 mg daily; *n* = 20), 6 patients developed thrombosis in the VKA-alone group compared with none in the HCQ plus VKA group [45]. In another small study that included 50 patients with APS, the use of HCQ as an adjunct treatment with anticoagulation achieved a lower risk of thrombosis at 2.6 years (0.001 vs 0.007; log-rank *P* = .048) [46]. However, following adjustment for covariates, the significant reduction in recurrent thrombosis was no longer present.

HCQ is generally well tolerated, and common side effects are mild (gastrointestinal upset, headaches, and rash). As long-term HCQ treatment can lead to retinal toxicity, patients on HCQ for greater than 5 years should be monitored for retinopathy [47]. However, annual monitoring is required for patients with additional risk factors for retinal toxicity, such as impaired renal function, concomitant tamoxifen use, or a dose of HCQ > 5 mg/kg/d [47]. As there are no large studies assessing the role of HCQ in preventing recurrent thrombosis in patients with APS, more evidence is required before it is recommended in routine clinical practice. However, European Medicine Agency has licensed HCQ as an orphan medicinal product in APS patients with refractory or recurrent thrombosis despite adequate anticoagulation due to its low-risk profile, no additional risk of bleeding, and promising results from early human studies [48].

A very small proportion of patients with thrombotic APS can develop recurrent thrombosis even after intensification of the INR to 3.5 (3.0-4.0) or the addition of antiplatelet treatment to standard-intensity INR. These patients require individualized care in a specialist center with expertise in the management of complex patients with APS as the options are limited and need to balance prevention of recurrence thrombosis vs increased risk of bleeding. Suggested options are addition of an antiplatelet agent to intensify the INR, immunomodulation with HCQ, or substituting warfarin with high-intensity LMWH (maintaining peak anti-Xa levels at 1.6-2.0 U/mL for once-daily dosing and peak at 0.8-1.0 U/mL for twice-daily dosing) [28,29]. In addition to HCQ, immunomodulatory agents such as rituximab (chimeric anti-CD20 monoclonal antibody), which has been shown to produce variable responses in reducing aPL levels in patients with APS, complement inhibitors such as eculizumab and mTOR inhibitors such as sirolimus, could be considered [28,29]. Our approach to the management of recurrent thrombosis in APS is summarized in the Figure.

**Figure:**
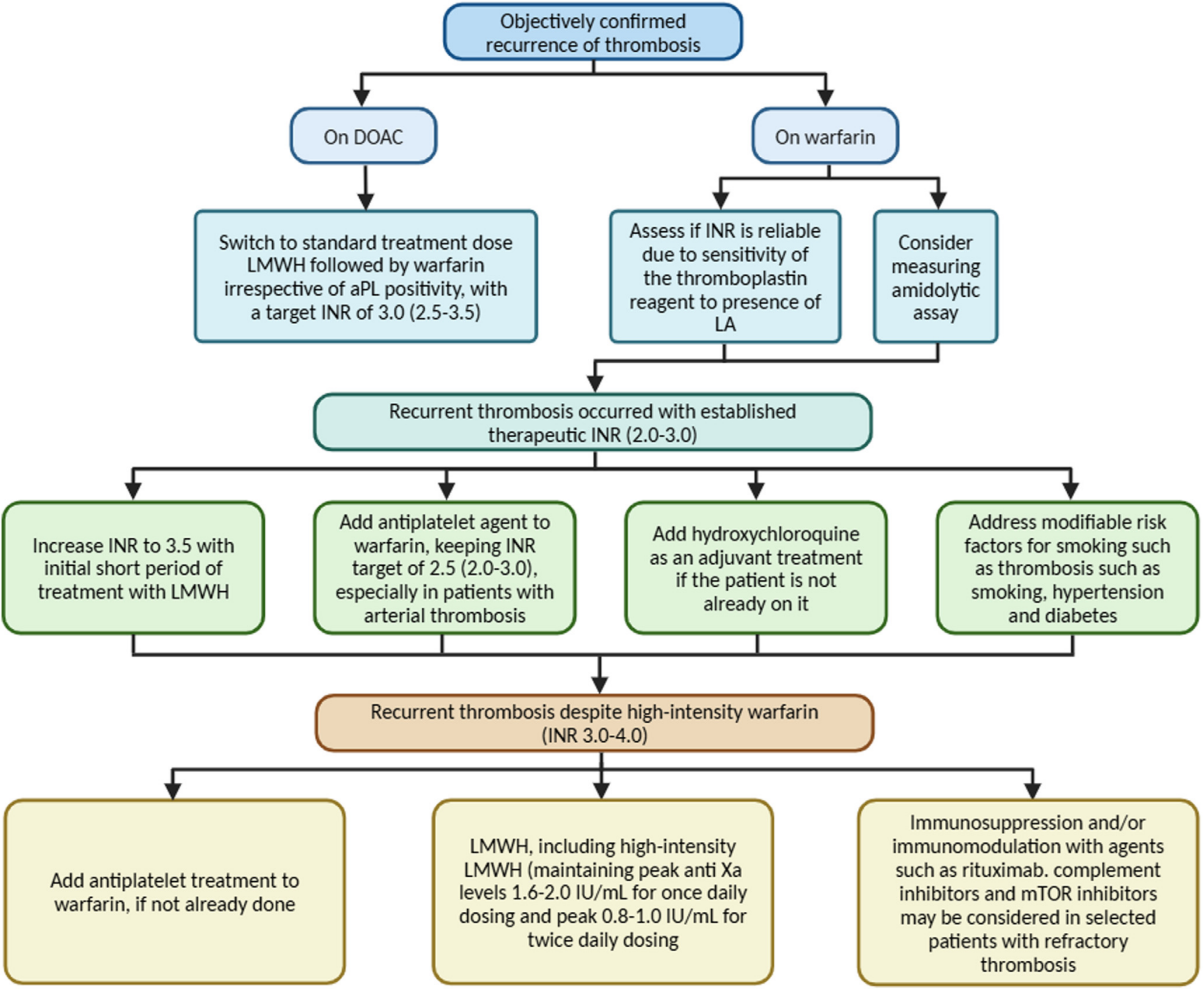
Suggested pragmatic approach to the management of recurrent thrombosis in patients with antiphospholipid syndrome. aPL, antiphospholipid antibody; DOAC, direct oral anticoagulant; LA, lupus anticoagulant; INR, international normalized ratio; LMWH, low-molecular-weight heparin.

## 7 What to do with Fluctuating Levels of Antibodies or Alternatively Positive aPL and a History of Thrombosis

The link between aPL and pathogenesis of thrombosis is well established, although some of the pathogenic mechanisms remain uncertain. Some patients are reported to change their LA, aCL, or anti-β2GPI status from positive to negative or show fluctuation of the antibody level over the course of their disease following the original confirmatory tests. It is an unresolved question whether disappearance of aPL means that there is no ongoing risk of developing recurrent thrombosis. It is very important to establish whether testing methods and the reference material have changed when interpreting the negative or positive results. Furthermore, APS ACTION International Clinical Database and Repository assessed 230 patients with APS in 4 consecutive yearly samples for fluctuation of aPL titers and found that they may decrease at the time of thrombosis [49]. Additionally, as APS is an autoimmune disease, aPL titers may increase during acute infection or inflammation, as was observed during COVID-19. Interpretation of LA needs even further caution as all anticoagulants can affect LA testing, most frequently causing false-positive LA results unless appropriate precautions such as a DOAC stop test are used [[50], [51], [52]]. Mixing studies of patients on warfarin, where patient plasma is diluted with pooled normal plasma, is not advisable since a weakly positive LA can be missed [[53], [54], [55]]. At present, there is insufficient evidence to suggest that stopping anticoagulation is safe in patients with thrombosis and a previously confirmed diagnosis of APS. Therefore, in the absence of strong risk factors for major bleeding, we suggest continuing anticoagulation in patients with a confirmed diagnosis of thrombotic APS but fluctuation of aPL.

Some patients with thrombosis have alternating positive aPL (eg, positive LA, which becomes negative but subsequently positive for aCL or anti-β2GPI). Based on the classification criteria for APS, patients with fluctuating positive alternate antibodies do not have APS. In practice, we may consider them as single positive aPL associated with thrombosis, and as discussed earlier, there is insufficient evidence to make a definite recommendation on the use of VKA over DOAC in these patients. Therefore, in this situation, we either use standard dose DOAC or VKA with a target INR of 2.5 (2.0-3.0) based on the patient’s preference and clinical history.

## 8 Management of Asymptomatic Carriers

Another very important unresolved issue frequently presented to clinicians is the significance of incidentally detected aPL in individuals with no history of clinical events. Specifically, should primary thromboprophylaxis be offered to individuals with persistently positive aPL, especially those with triple-positive aPL (aPL carriers)? Two older studies found that asymptomatic individuals with aPL did not have an increased risk of thrombosis [56,57]. However, in these studies, asymptomatic individuals received thromboprophylaxis with LMWH or aspirin for the periods when they were at increased risk of thrombosis, such as following surgery or immobilization. Furthermore, counseling and treatment for vascular risk reduction (diabetes, smoking, hypertension, and obesity) were offered to patients with APS and asymptomatic individuals with aPL [56,57]. Therefore, at present, we do not know whether these patients would be at high risk of thrombosis without these interventions.

More recent studies indicated that aPL carriers may have increased risk of thrombosis, especially those with triple-positive aPL [58,59]. In a multicenter study that included 104 subjects with a high-risk aPL profile (triple-positive), an annual first thrombosis rate of 5.3% was observed, with a roughly equal split between venous and arterial events. These subjects did not receive long-term routine thromboprophylaxis but received short-term thromboprophylaxis with once-daily LMWH when exposed to high risk of thrombosis, such as surgery or prolonged immobilization. The 10-year cumulative thrombosis incidence was 37.1% [58]. However, it is not possible to exclude selection bias and ensure the study population is a genuine representative cohort of the general population.

In a meta-analysis including 1208 individuals from observational studies, it was found that there was a reduction in arterial, but not venous, thrombotic events for aPL carriers treated with LDA (OR, 0.48; 95% CI, 0.28-0.82) [60]. In 2017, in a study that included 98 individuals, a 2.3% first thrombotic rate was observed per year, with a stronger association with triple-positive aPL [59]. In 2018, a Cochrane review, which included 1044 patients randomized to various primary thromboprophylaxis strategies, concluded that there was insufficient evidence to determine the benefit or harm of using anticoagulation or aspirin alone or in their combination in preventing thrombosis in aPL carriers [61]. However, in a mixed population of people with a history of miscarriages who received a combination of anticoagulation and acetylsalicylic acid, the incidence of minor bleeding (nasal bleeding and menorrhagia) was increased when compared with acetylsalicylic acid alone [61]. Overall, the issue regarding primary thromboprophylaxis in persistently positive aPL carriers remains unresolved. British Society for Haematology guidelines [22] recommend anticoagulant thromboprophylaxis in individuals with asymptomatic aPL only during the periods of increased risk, such as postoperatively, including advice on vascular risk reduction with lifestyle modification such as regular exercise, healthy diet, and smoking cessation with avoidance of systemic estrogen therapies. However, European guidelines recommend offering prophylaxis with LDA to triple-positive aPL carriers [62]. There is no consensus on whether to offer thromboprophylaxis to individuals with persistently positive aPL during long-haul flights. It is our practice to offer thromboprophylaxis with LMWH to these individuals, especially those with triple-positive aPL, prior to each leg of the journey with good hydration and moving as much as they can during the flight.

## 9 Diagnosis and Management of CAPS

Although it is extremely rare, diagnosis and management of CAPS remains one of the most challenging issues in APS. CAPS was first recognized as a syndrome more than 30 years ago, but it is rare, and although an international registry has accumulated more than 700 cases, most of the literature is comprised of case reports, often with specific associations such as HIT or thrombopoietin receptor agonist therapy, which makes it difficult to generalize diagnosis and management and to estimate the true frequency or incidence [63]. The situation is not dissimilar from uncomplicated APS: there are criteria for classification that are frequently used for diagnosis but do not include all cases and leave a category of “probable CAPS” who nonetheless will need urgent treatment. Similarly, the presence of aPL is essential, but there are many noncriteria antibodies that may merit consideration when making a diagnosis.

Diagnosis of CAPS is complicated by its similarities and overlap with other acute systemic inflammatory disorders such as spontaneous HIT, VITT-like syndrome, hemophagocytic lymphohistiocytosis, macrophage activation syndromes, and acute sepsis, particularly when disseminated intravascular coagulation and microvascular thrombosis are present. Separation from these disorders remains a central problem in CAPS. Rapid identification of aPL, ideally with defined specificities combined with a broader diagnostic test pattern, remains an unfulfilled need.

### 9.1 Can CAPS be predicted or prevented?

Given its catastrophic nature, it would be highly valuable if CAPS could be predicted and prevented. Unfortunately, CAPS is the presenting feature of APS in about 50% of cases, but prevention might focus on several areas.

#### 9.1.1 Are some people genetically predisposed to CAPS?

Chaturvedi et al. [64] demonstrated complement activation in 85.7% of CAPS patients and went on to show that they have rare germline variants in complement regulatory genes more frequently (60%) than those with APS (21.8%), systemic lupus erythematosus (28.6%), or normal controls (23.3%). The value of this observation is that, besides identifying high-risk individuals, it indicates that complement inhibition may be beneficial, at least for some patients, both acutely and in the long term.

Some authors have suggested that thrombophilic traits might also be a predisposing factor. In an individual with an underlying heritable thrombophilic defect, the presence of aPL may synergistically act to increase the risk of developing CAPS compared with single-site thrombosis in uncomplicated APS.

Do some APS patients have antibody profiles that are more likely to produce CAPS? From the severity of the syndrome, it might be expected that all CAPS would be triple positive, but this is not the case [65].

#### 9.1.2 Are there some circumstances (second hits) that are likely to precipitate CAPS?

Candidates for “second hits” abound, and this is reflected in the high number of case reports. Prominent among these is infection, and as with other disorders (eg autoimmune heparin induced thrombocytopenia and thrombosis), it is postulated that the cross-reaction of antibodies due to the similarity in infectious and endogenous antigens may trigger the syndrome. Sepsis is the reported trigger in 49% of cases [65]. However, at present, the associations are too diverse and too prevalent to be useful in prediction.

#### 9.1.3 Anticoagulation

It has been suggested that more rigorous attention to maintenance of anticoagulation may reduce the risk of CAPS in high-risk situations such as surgery [66]. The authors found an excess of patients who had a subtherapeutic INR or were otherwise not optimally anticoagulated in the CAPS group compared with non-CAPS patients with APS. Although anticoagulation is clearly important, prospective data are required to establish benefit of preventing CAPS.

### 9.2 Management of CAPS

For many years, the basic recommendations for management have comprised anticoagulation, plasma exchange, and corticosteroids based on reports in the CAPS registry [65,67]. In the acute management of CAPS, anticoagulation with unfractionated heparin should be monitored using heparin anti-Xa levels rather than activated partial thromboplastin time, as patients with CAPS have elevated baseline activated partial thromboplastin time [22]. In some cases, the addition of intravenous immunoglobulin has also been recommended. As with APS, an increased understanding of mechanisms should facilitate more targeted therapies. Indeed, more specific immunosuppression with agents such as rituximab [68] or sirolimus [69] and blocking of the complement pathways [70] are reported as successful. An unanswered question is whether these should join or even replace the existing recommendations, but again, the heterogeneity of APS is a barrier to implementation, and responses to eculizumab, for example, are inconsistent [71,72]. How we can identify which patients will benefit from these additional treatments remains an unresolved issue.

## 10 Conclusions

APS is a highly heterogeneous antibody-mediated autoimmune disease. Although classification criteria for APS have been developed, there are no standardized diagnostic criteria for APS. The diagnosis of APS in routine clinical practice is challenging due to many overlapping factors that can contribute to thrombosis in patients with aPL. Despite its autoimmune nature, secondary thrombosis prevention is achieved by anticoagulation, mainly with VKA, such as warfarin, rather than immunomodulation. Although evidence is convincing for the use of VKA in triple-positive APS with VTE, further evidence is required for the unequivocal recommendation on the use of VKA in patients with VTE and single or dual positive aPL, although it is suggested to use VKA in such patients. Management of patients with stroke/arterial thrombosis with aPL remains a major unresolved issue, although our current British Society for Haematology guidelines recommend use of warfarin as the first-line treatment for stroke in APS. The role of immunomodulation in thrombotic APS certainly needs further investigation, as such an approach would target the pathogenic mechanisms and reduce the risk of bleeding that is associated with anticoagulants. Recurrent thrombosis, despite being on treatment dose anticoagulation, may be explained by thrombo-inflammation. Therefore, further attention to modifying thrombo-inflammation is required in future studies. It is not clear whether stopping anticoagulation is safe in a patient who had previously confirmed thrombotic APS but now has negative aPL. The best approach in the management of CAPS remains an unresolved issue due to its rarity, and conducting clinical studies is extremely challenging.
